# IL-17A is essential to the development of elastase-induced pulmonary inflammation and emphysema in mice

**DOI:** 10.1186/1465-9921-14-5

**Published:** 2013-01-20

**Authors:** Etsuko Kurimoto, Nobuaki Miyahara, Arihiko Kanehiro, Koichi Waseda, Akihiko Taniguchi, Genyo Ikeda, Hikari Koga, Hisakazu Nishimori, Yasushi Tanimoto, Mikio Kataoka, Yoichiro Iwakura, Erwin W Gelfand, Mitsune Tanimoto

**Affiliations:** 1 Department of Hematology, Oncology, Allergy and Respiratory Medicine, Okayama University Graduate School of Medicine, Dentistry, and Pharmaceutical Sciences, Okayama, Japan; 2 Center for Experimental Medicine and Systems Biology, Institute of Medical Science, University of Tokyo, Tokyo, Japan; 3 Division of Cell Biology, Department of Pediatrics, National Jewish Health, Denver, CO, USA

**Keywords:** IL-17, Elastase, Emphysema, Chronic obstructive pulmonary disease

## Abstract

**Background:**

Pulmonary emphysema is characterized by alveolar destruction and persistent inflammation of the airways. Although IL-17A contributes to many chronic inflammatory diseases, it’s role in the inflammatory response of elastase-induced emphysema remains unclear.

**Methods:**

In a model of elastase-induced pulmonary emphysema we examined the response of IL-17A-deficient mice, monitoring airway inflammation, static compliance, lung histology and levels of neutrophil-related chemokine and pro-inflammatory cytokines in bronchoalveolar lavage (BAL) fluid.

**Results:**

Wild-type mice developed emphysematous changes in the lung tissue on day 21 after elastase treatment, whereas emphysematous changes were decreased in IL-17A-deficient mice compared to wild-type mice. Neutrophilia in BAL fluid, seen in elastase-treated wild-type mice, was reduced in elastase-treated IL-17A-deficient mice on day 4, associated with decreased levels of KC, MIP-2 and IL-1 beta. Elastase-treated wild-type mice showed increased IL-17A levels as well as increased numbers of IL-17A+ CD4 T cells in the lung in the initial period following elastase treatment.

**Conclusions:**

These data identify the important contribution of IL-17A in the development of elastase-induced pulmonary inflammation and emphysema. Targeting IL-17A in emphysema may be a potential therapeutic strategy for delaying disease progression.

## Background

Chronic obstructive pulmonary disease (COPD) is characterized by progressive alveolar destruction and inflammation of the airways caused by noxious particles or gases. The precise mechanisms behind these pathological changes remain unclear, although oxidative stress, elastinolytic activity, and apoptosis of lung parenchymal cells are believed to contribute to the pathogenesis of the disease [1]. Current anti-inflammatory therapies are poorly effective in maintaining lung function and symptoms in COPD [2], therefore, new anti-inflammatory therapeutic strategies are needed.

It has been reported that there are increased numbers of tertiary lymphoid follicles in the lungs of patients with COPD [3,4], and that these follicles are organized structures with a B-cell core, T cells in the periphery, and dendritic cells capable of antigen presentation [5-7]. Such observations have led to the hypothesis that COPD has an autoimmune component [8-10].

IL-17 is produced by inflammatory cells and targets structural cells such as epithelial cells, fibroblasts, and smooth muscle cells. The IL-17 family comprises six members (IL-17A to IL-17 F). IL-17A and IL-17 F display high sequence homology and can be secreted as homodimers, as well as IL-17A/F heterodimers, by both mouse and human cells [11]. These cytokines are produced by a subset of CD4+ lymphocytes known as T-helper (Th) 17 cells [12] and contribute to the development of autoimmune disease [13,14]. IL-17A has been shown to induce the production of chemokines and inflammatory cytokines such as keratinocyte-derived chemokine (KC), macrophage inflammatory protein 2 (MIP-2), and IL-1β, that promote neutrophilic inflammation [15-17].

Recently, IL-17 has been shown to be associated with the development of lung inflammatory diseases such as COPD [16,18,19]. Production of IL-17A was increased in the bronchial submucosa and subepithelium of COPD patients [18,19]. Airway smooth muscle strips from COPD patients expressed IL-17RA and responded to IL-17 by inducing IL-8 production [16]. However, other studies failed to show any involvement of IL-17 in the development of COPD. Numbers of IL-17A+ cells in the airways of COPD patients were increased compared to healthy control subjects, but were not increased compared to smoking control subjects, and were not associated with increased neutrophilic inflammation [20]. Expression of IL-17 receptors in COPD was not different compared to non-COPD lung disease [21].

Nonetheless, the role of IL-17A in the pathogenesis of COPD remains controversial and often has been based on indirect evidence. To address this question, we instilled porcine pancreatic elastase (PPE) in the trachea which first induces lung inflammation and subsequently results in alveolar wall destruction [22,23]. These pathological changes closely mimic those seen in human emphysema. Here, we investigated the role of IL-17 in the development of elastase-inducible emphysema using IL-17A-deficient (IL-17A−/−) mice.

## Methods

### Animals and treatment

C57BL/6 wild-type (WT) mice were obtained from Charles River Japan. IL-17A−/− mice were generated and backcrossed to C57BL/6 mice for 10 generations [24]. Ten week-old female WT and female IL-17A−/− mice were used in experiments.

WT and IL-17A−/− mice received 3.5u of PPE (Sigma-Aldrich, St. Louis, MO, USA) in 30 μl of saline by intratracheal instillation on day 0 after being anesthetized with isoflurane. As control groups, WT and IL-17A−/− mice received 30 μl of saline on day 0. To evaluate emphysematous changes in the lungs, lung function was measured, and lung histology and morphometric measurements of air space size were assessed 21 days after PPE administration. On days 2, 4, and 21 after PPE administration, lungs were lavaged for analysis of cellular composition in bronchoalveolar lavage (BAL) fluid.

To determine the timing of production of IL-17A in the airways, WT mice received PPE, and then lung tissue was taken at different time points (12 hours, 1 day, 2 day, 4 day, and 21 days) after PPE administration, and the levels of IL-17A in lung homogenates were measured.

To assess the numbers of IL-17A and IFN-γ producing cells, WT mice received PPE or saline on day 0, and lung cells were isolated and IL-17A-producing cells were assessed by flow cytometry on day 2 after PPE administration.

All experiments were performed in accordance with the National Institutes of Health guidelines. All protocols were approved by the Institutional Animal Care and Use Committees of Okayama University (Okayama, Japan).

### Determination of static lung compliance (Cst)

21 days after PPE administration, lung function was measured in tracheostomized mice using a computer-controlled small-animal ventilator (flexiVent; SCIREQ, Montreal, Canada) as described previously [25-27]. In brief, the mice were anesthetized with sodium pentobarbital (Kyoritsu Seiyaku, Tokyo, Japan) by intraperitoneal injection (12.5 mg/kg = 250 μl/mouse). The mice were tracheostomized with a 5 mm section of metallic tubing (= 18 G cannula), ventilated at 180 breaths/min with a tidal volume of 4 ml/kg and positive end-expiratory pressure of 3 cm H_2_O, and Cst was assessed. All data points were determined by flexiVent software (version 5.0).

### Lung histology and morphometric measurements of air space size

On day 21 after PPE administration, mice were killed by intraperitoneal injection of pentobarbital sodium and lungs were inflated and fixed by intratracheal instillation of 10% formalin at a constant pressure of 25 cmH_2_O. The tissue was then embedded in paraffin and 2-μm thick sections were stained with hematoxylin and eosin (H&E). Air space enlargement was quantified by mean linear intercept (Lm) calculations in 20 randomly selected fields [28].

### BAL

On days 2, 4, and 21 after PPE administration, lungs were lavaged via the tracheal tube with Hanks’ balanced salt solution (2 × 1 ml, 37°C). The volume of the collected BAL fluid was measured in each sample, and the number of cells in the BAL fluid was counted. Cytospin slides were stained and differentiated in a blinded fashion by counting at least 200 cells under light microscopy [29].

### Measurement of cytokines and chemokines

KC, MIP-2 and IL-1β levels in the BAL fluid, and IL-17A levels in the supernatants of homogenized lungs were measured by ELISA as previously described [30]. For preparation of lung homogenates, lung tissue was frozen at −70°C immediately after euthanasia. Lung tissue was mixed with a PBS-0.1% Triton-X100 solution containing proteinase inhibitors at a 1:2.5 ratio of weight per volume (BD Biosciences, San Diego, CA, USA). The specimens were homogenized and then centrifuged at 15,000 rpm for 15 min. The supernatants were frozen at −70°C until analysis [30]. All cytokine and chemokine ELISAs were performed according to the manufacturer’s directions (R&D Systems, Minneapolis, MN, USA). The limits of detection were 5 pg/ml for IL-17A, 2 pg/ml for KC, 1.5 pg/ml for MIP-2, and 3 pg/ml for IL-1β.

### Flow cytometry

Lung cells were isolated as previously described following collagenase digestion [30]. Intracytoplasmic cytokine staining was performed as previously described [31,32]. Briefly, after purification, cells were stimulated in vitro with phorbol myristate acetate (Sigma-Aldrich) and ionomycin (Sigma-Aldrich). Cells were stained for cell surface markers with PerCP-conjugated anti-CD4 (BD Biosciences). Then, cells were stained with PE-conjugated anti-IL-17A (BD Biosciences) or FITC-conjugated anti-IFN-γ (BD Biosciences), and analyzed on MACS Quant flow cytometer (Miltenyi Biotec, Auburn, CA, USA) with FlowJo software (TreeStar, Ashland, OR, USA). The numbers of cytokine-producing CD4 T cells per lung were calculated from the percentages of cytokine-producing cells and the numbers of CD4 T cells isolated from the lung.

### Statistical analysis

All results were expressed as the means ± standard error of the means (SEM). Statistical significance was detected by using 1-way ANOVA, followed by post-hoc analysis with the Tukey-Kramer test for comparisons between multiple groups. As measured values may not be normally distributed due to the small sample sizes, nonparametric analysis, Mann–Whitney *U*-test, was used for comparison of the numbers of cytokine-producing cells. Significance was assumed at p values of < 0.05.

## Results

### Development of emphysema is reduced in IL-17A−/− mice

**Figure 1:**
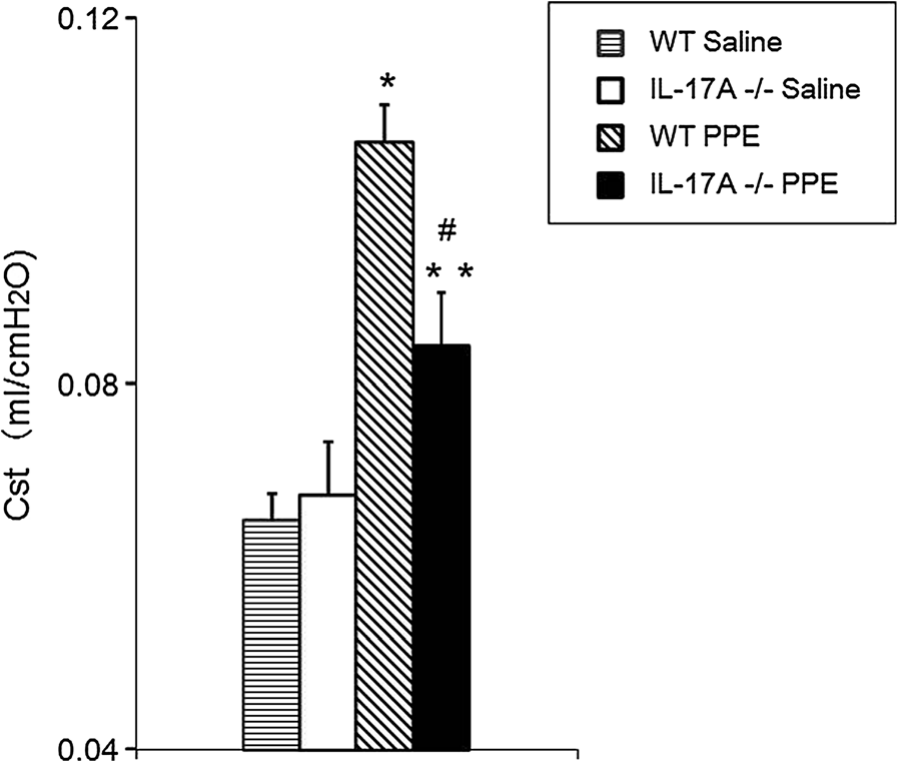
**Static lung compliance (Cst) values on day 21 after intratrachial instillation of porcine pancreas elastase (PPE) or saline.** The results are expressed as means ± SEM (n=12 in each group). *p<0.01 compared to WT saline and IL-17A−/− saline. **p<0.05 compared to WT saline and IL-17A−/− saline. #p<0.01 versus WT PPE.

To elucidate the role of IL-17A in the development of emphysema, we examined lung function 21 days following PPE treatment. Cst values were significantly higher in PPE-treated WT mice (mean±SEM: 0.106 ± 0.004 ml/cmH2O) compared with the values in saline-treated WT mice (0.065 ± 0.003 ml/cmH2O) and IL-17A−/− mice (0.067 ± 0.005 ml/cmH2O) 21 days after treatment (Figure 1). PPE-treated IL-17A−/− mice developed only small increases in Cst (0.084 ± 0.005 ml/cmH2O) above saline-treated control mice (p <0.05), but the values were significantly lower than demonstrated in PPE-treated WT mice (p<0.01).

**Figure 2:**
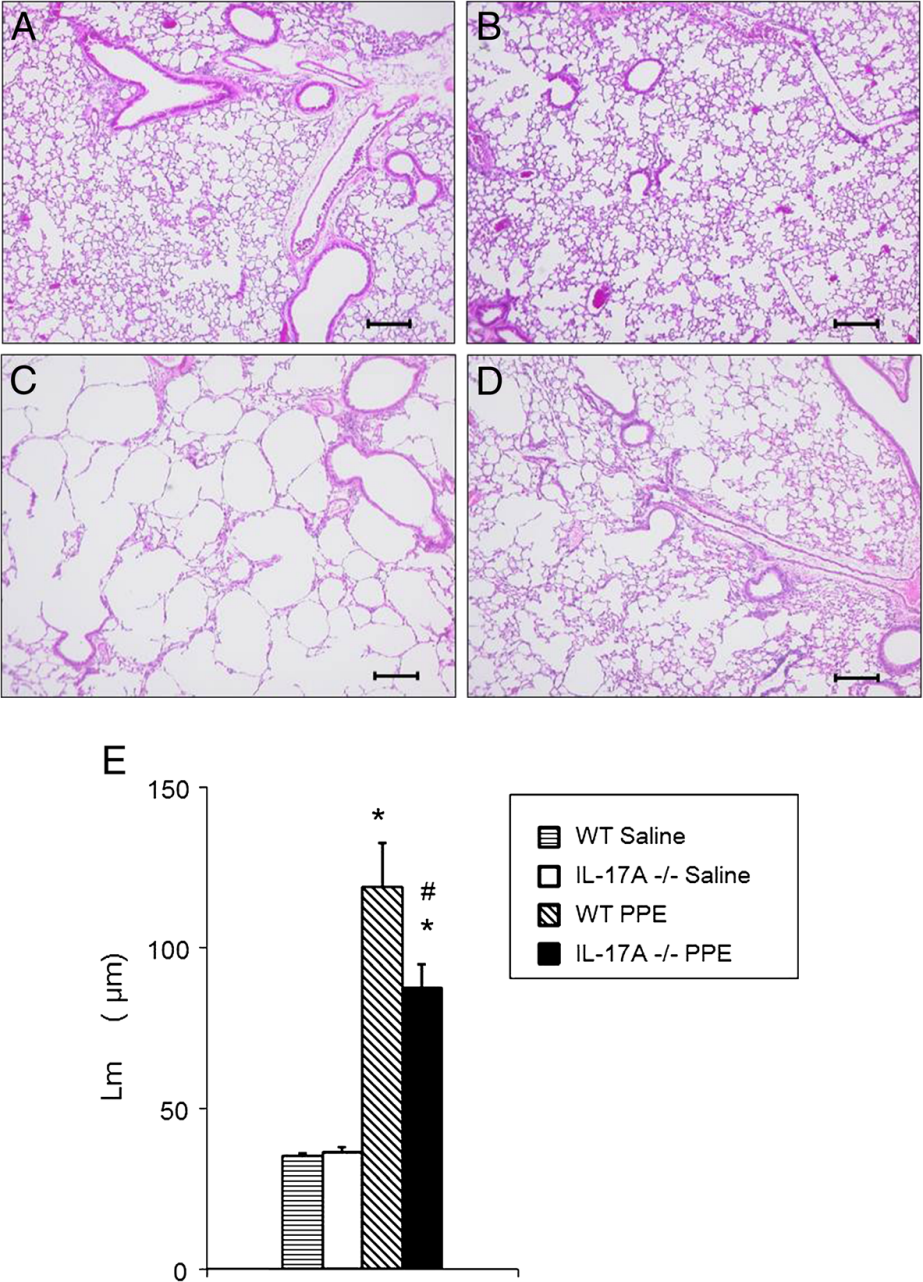
**Morphometric assessment performed on day 21 after instillation of PPE or saline.** Representative photographs of lungs of WT mice treated with saline **(A)**, IL-17A−/− mice treated with saline **(B)**, WT mice treated with PPE **(C)**, and IL-17A−/− mice treated with PPE **(D)** groups. **(E)** Mean linear intercepts (Lm) values of alveoli. Lm values were obtained as described in Methods. Bar = 200 μm. Data represent the mean ± SEM (n=12 in each group). *p<0.01 compared to WT saline and IL-17A−/− saline. #p<0.05 versus WT PPE.

Lung tissue was stained with H&E. No pathological differences were observed in WT and IL-17A−/− mice after saline treatment upon microscopic examination (Figure 2A and B). Intratracheal PPE instillation to WT mice produced air space enlargements and alveolar wall destruction (Figure 2C) and these pathological changes were much less severe in IL-17A−/− mice. As seen in Figure 2D, the alveolar walls were less severely damaged in the lungs of IL-17A−/− mice treated with PPE compared to WT mice.

To further characterize the development of emphysema in IL-17A−/− mice, we determined the airspace enlargement by measuring the mean linear intercept (Lm) in 20 randomly selected fields from H&E stained tissue sections. After PPE treatment, Lm values in WT mice (118.6 ± 13.9 μm) were significantly increased compared to saline-treated WT mice (35.1 ± 0.9 μm) and IL-17−/− mice (36.5 ± 1.5 μm) (Figure 2E). The increases in Lm values were significantly reduced in IL-17A−/− mice (87.5 ± 7.4 μm) compared to WT mice 21 days after treatment with PPE (Figure 2E). These histological and physiological evaluations demonstrated that IL-17A−/− animals were less susceptible to PPE-induced changes in the lung.

### Elastase-induced pulmonary inflammation is reduced in IL-17A −/− mice

**Figure 3:**
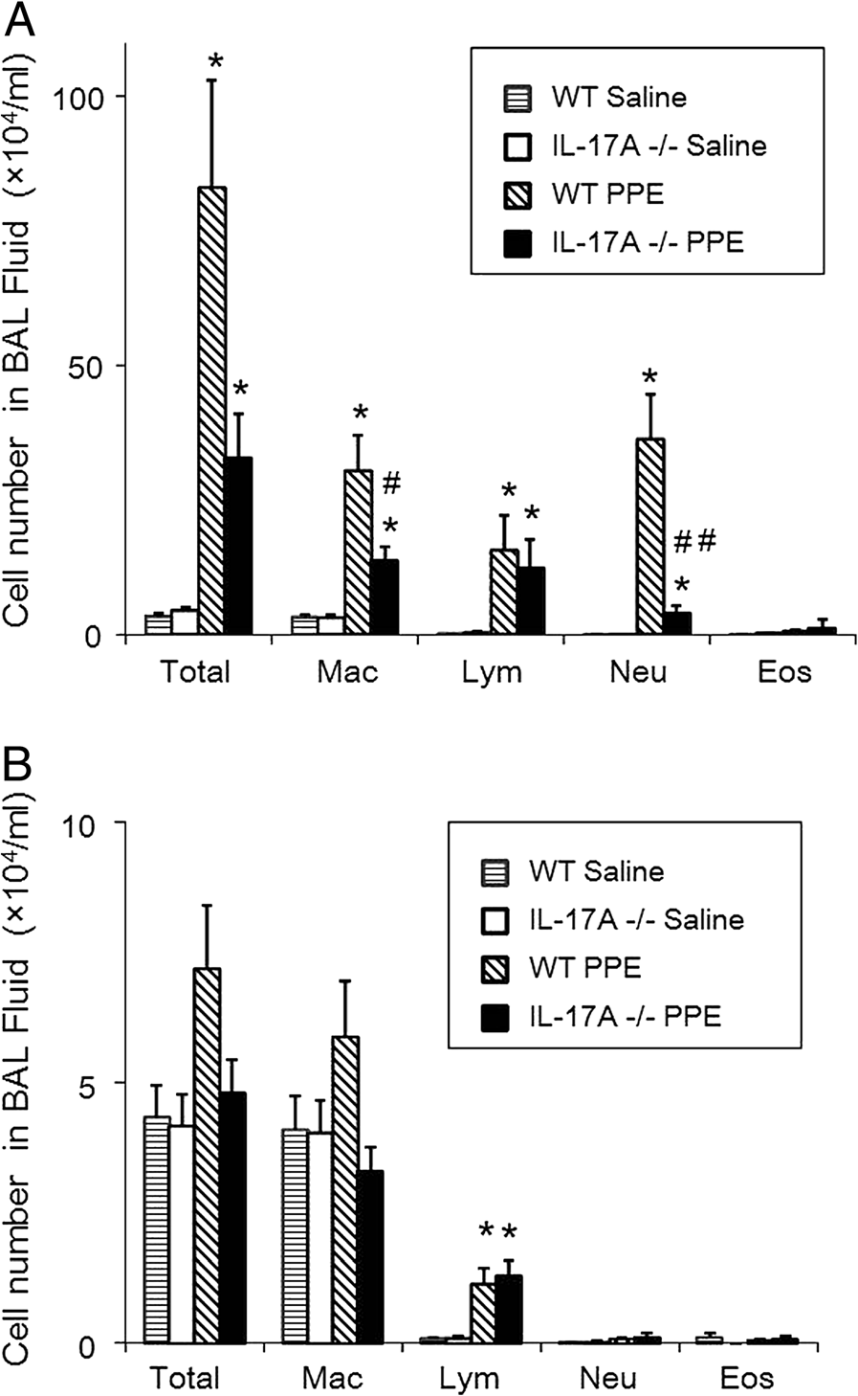
**Cellular composition in bronchoalveolar lavage (BAL) fluid after intratracheal instillation of PPE or saline.** BAL fluid was obtained as described in Methods. Data represent the mean ± SEM (n=12 in each group). **(A)** Cellular composition in BAL fluid on day 4 after intratracheal instillation of PPE or saline. *p<0.01 compared to WT saline and IL-17A−/− saline. #p<0.05 versus WT PPE. ##p<0.01 versus WT PPE. **(B)** Cellular composition in BAL fluid on day 21 after intratracheal instillation of PPE or saline. *p<0.05 compared to WT saline and IL-17A−/− saline.

Early onset lung inflammation is thought to be a major contributor to the development of emphysema [33]. The severity of the initial inflammation was compared in WT and IL-17A −/− mice 4 days after PPE treatment. In WT mice, inflammatory cell recruitment into the airways was increased after PPE treatment (Figure 3A). Increased total cell numbers were largely due to increased numbers of neutrophils and macrophages in BAL fluid (Figure 3A). At this early stage, few histological changes were detected (data not shown). The increases in neutrophil numbers were significantly lower in IL-17A−/− mice compared to WT mice 4 days after PPE treatment (41.2 ± 5.4 vs 5.2 ±1.1 ×10^4^/ml) (Figure 3A).

We also assessed inflammatory cell population in BAL fluid 21 days after PPE treatment when emphysematous changes were already established. Numbers of lymphocytes in PPE-treated WT and IL-17A−/− mice were higher compared to saline-treated WT and IL-17A−/− mice (Figure 3B). However, no differences were observed between PPE-treated WT mice and PPE-treated IL-17A−/− mice in cellular composition at this point in time (Figure 3B).

### Cytokine and chemokine levels in BAL fluid

**Figure 4:**
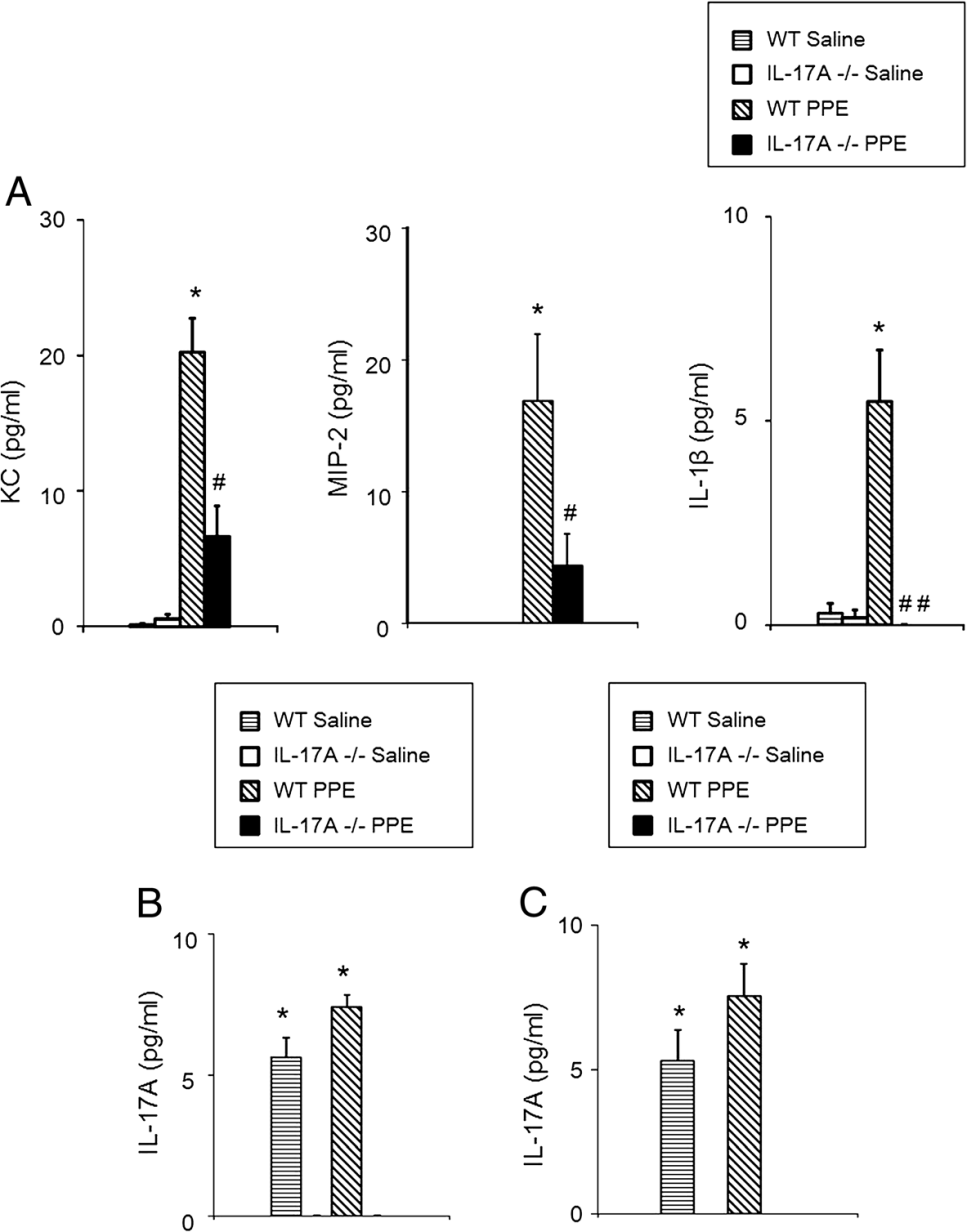
**Cytokine and chemokine levels in the BAL fluid and lung homogenates. (A)** KC, MIP-2, and IL-1β levels in BAL fluid. BAL fluid was obtained from the same groups as described in Figure 3A. Data represent the mean ± SEM (n=12 in each group). *p<0.01 compared to WT saline and IL-17A−/− saline. #p<0.05 versus WT PPE. ##p<0.01 versus WT PPE. **(B)** IL-17A levels in lung homogenates on day 4 after intratracheal instillation of PPE or saline. Data represent the mean ± SEM (n=8 in each group). **(C)** IL-17A levels in lung homogenates on day 21 after intratracheal instillation of PPE or saline. Data represent the mean ± SEM (n=8 in each group).

To determine the basis for the altered inflammatory cellular recruitment in the IL-17A−/− mice, we assessed levels of BAL neutrophil-related chemokines (KC, MIP-2) and pro-inflammatory cytokine (IL-1β) 4 days after PPE treatment. KC, MIP-2, and IL-1β levels were all higher in WT mice treated with PPE compared to mice which received saline (Figure 4A). Levels of KC, MIP-2, and IL-1β in BAL fluid of IL-17A−/− mice were significantly lower compared to WT mice following PPE treatment. We next assessed IL-17A levels in lung homogenates because it could not be detected in BAL fluid. Levels of IL-17A in lung homogenate of PPE-treated WT mice tended to be higher compared to saline-treated WT mice, but were not significantly different (7.5 ± 0.5 and 5.4 ± 0.4 pg, respectively) (Figure 4B).

We also assessed chemokine and cytokine levels in BAL fluid on day 21 after PPE or saline treatment. At this point in time, KC, MIP-2, and IL-1β levels were all lower than limits of detection. Levels of IL-17A in lung homogenates of PPE-treated WT mice also were not higher compared to saline-treated WT mice (7.3 ± 1.0 and 5.3 ± 0.4 pg, respectively) (Figure 4C).

### Kinetics of IL-17A levels in the lung

**Figure 5:**
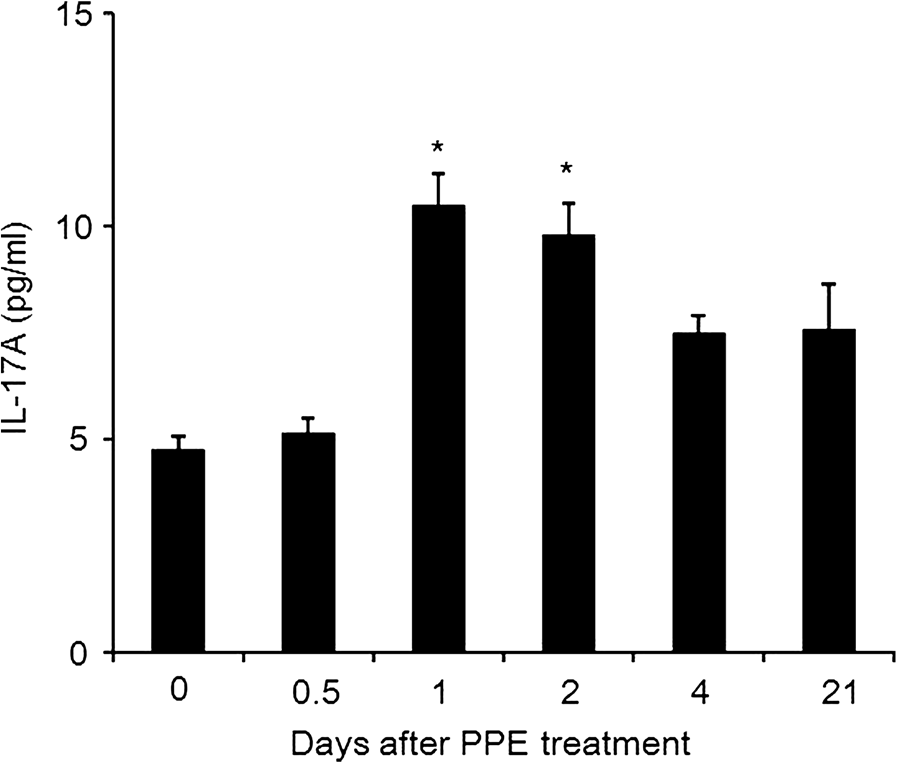
**IL-17A levels in the lung. IL-17A levels in lung homogenates of WT mice after intratracheal instillation of PPE were measured by ELISA as described in Methods.** The results for each group are expressed as means ± SEM (n=8 in each group). *p <0.01, day 0 and day 0.5 versus day 1 and day 2.

To determine the timing of production of IL-17A in the airways following PPE treatment, we measured the levels of IL-17A in lung homogenates from WT mice at different time points. IL-17A levels were significantly higher early after PPE, on days 1 and 2 (10.5 ± 0.7 and 9.8 ± 0.7 pg, respectively) compared to baseline (day 0) and 12 hours (4.8 ± 0.3 and 5.1 ± 0.4 pg, respectively) after PPE treatment (Figure 5). The levels of IL-17A on days 4 or 21 (7.6 ± 0.4 and 7.6 ± 1.1 pg, respectively) following PPE treatment were not significantly higher than levels at baseline (day 0).

### Numbers of Th17 cells in the lung

**Figure 6:**
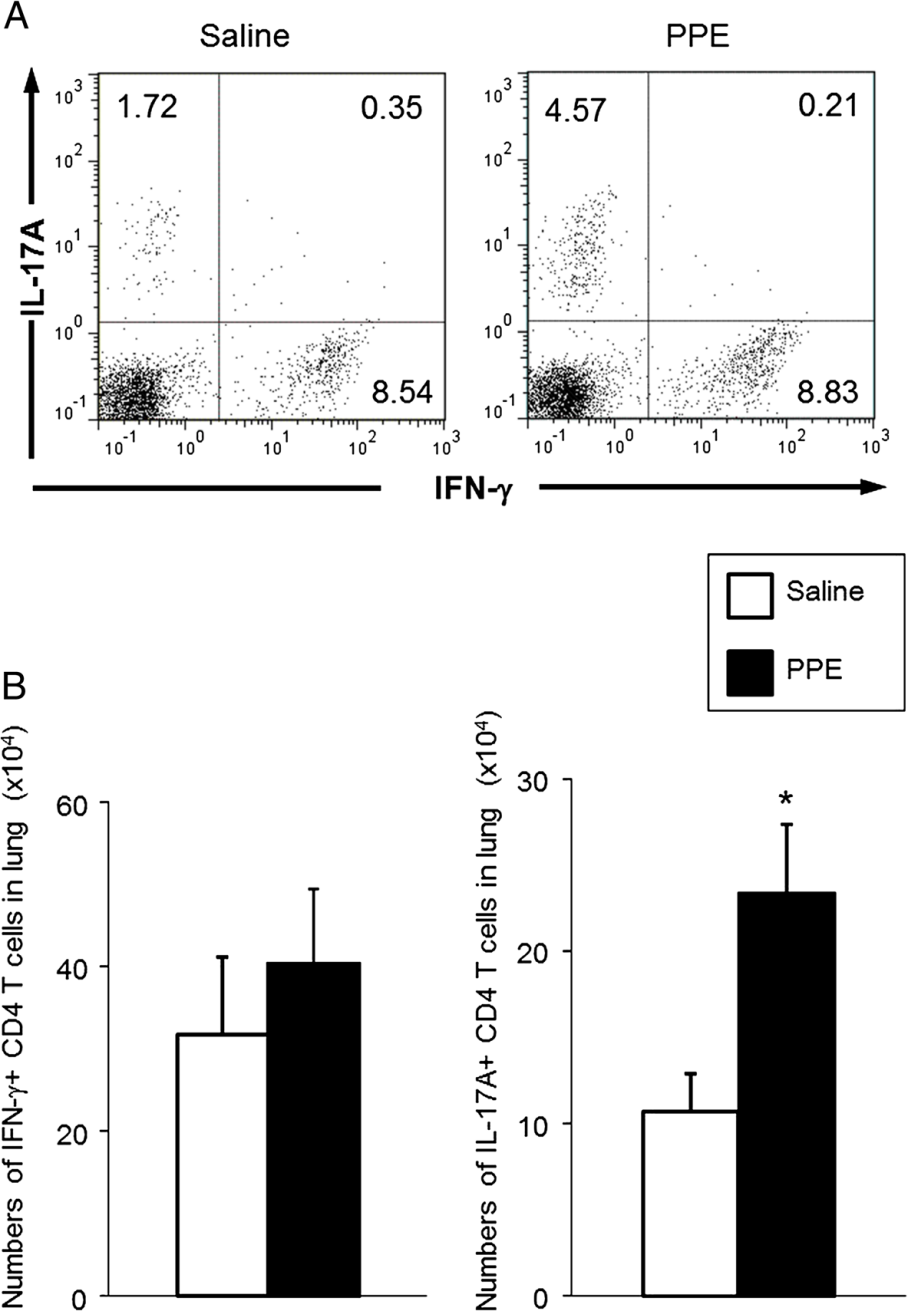
**Intracellular detection of IL-17A and IFN-γ in lung CD4+ T cells from WT mice 2 days after elastase administration. (A)** Percentages of IL-17A+ and IFN-γ+ T cells following saline treatment (Saline group) or PPE treatment (PPE group). Intracellular IL-17A and IFN-γ were assessed as described in Methods. The data shown are representative of two independent experiments. **(B)** The numbers of IL-17A+ and IFN-γ+ CD4 T cells in the lung of WT mice. The numbers of IL-17A+ CD4 T cells and IFN-γ+ CD4 T cells were calculated as described in Methods. Groups are the same as in **(A)**. Data represent the mean ± SEM (n=6 in each group). *p<0.05 versus saline-treated group.

We next determined the numbers of IL-17 producing cells in the lungs of WT mice, 2 days following elastase treatment. Previous studies have shown that Th1 cells contribute to the pathogenesis of COPD, therefore, we also determined IFN-γ producing cells [34,35]. Figure 6A shows the representative figures of percentages of IL-17A+ and IFN-γ+ CD4 T cells in the lung. IL-17A was detected in 4.78% of lung CD4 T cells following PPE treatment (4.57% for IL-17A+ IFN-γ-, 0.21% for IL-17A+ IFN-γ+), whereas it was detected in 2.07% (1.72% for IL-17A+ IFN-γ-, 0.35% for IL-17A+ IFN-γ+) following saline treatment. Figure 6B summarizes the numbers of cytokine-producing cells in the lungs from WT mice. The numbers of IL-17A+ CD4 T cells following PPE treatment were significantly higher compared to those following saline treatment (23.3 ± 4.0 vs 10.7 ± 2.2 ×10^4^, p<0.05), whereas the numbers of IFN-γ + CD4 T cells were not different between saline-treated WT mice and PPE-treated WT mice (40.4 ± 9.1 and 31.8 ± 9.3 ×10^4^, respectively). We assessed the cellular composition in BAL fluid of WT mice and IL-17A−/− mice on day 2 after PPE treatment. At this early stage, the numbers of neutrophils were significantly lower in IL-17A−/− mice compared to WT mice (attached file). These data suggest that elastase increases numbers of Th17 cells and secretion of IL-17A in the lungs, and this contributes to acute neutrophilic airway inflammation, and subsequently results in emphysematous changes in the airways.

## Discussion

It has been reported that Th17 cells are a critical component of the adaptive immune response and IL-17 has been implicated in chronic inflammatory and autoimmune diseases [13,14]. There has been much interest in the role of the adaptive immune response in COPD since COPD in humans is associated with the development of lymphoid follicles in the lung [3,4]. Moreover, patients with COPD have evidence of anti-elastin antibodies and Th1 responses, suggesting an autoimmune component [8,9]. Recently, numbers of IL-17A+ cells in the airways [18-20,36] and peripheral blood [37] of COPD patients have been reported to be increased. These studies suggested an association between Th17 and COPD, however, the role for IL-17A in disease development is not defined.

In the present study, to elucidate the role of IL-17A in the development of emphysema, we investigated PPE-induced initial inflammation and subsequent late phase emphysematous change in IL-17A−/− and WT mice. We demonstrated that both the PPE-induced acute phase inflammation and the late phase emphysematous changes seen in WT mice were attenuated in IL-17A−/− mice. Increased levels of neutrophil-related chemokines such as KC, MIP-2 and IL-1β seen in the BAL fluid of WT mice were also significantly reduced in IL-17A−/− mice following PPE administration. These data, for the first time, indicated that IL-17A may play a role in the elastase-induced pulmonary pathology.

We observed that levels of IL-17A in the lungs were increased in the acute phase following PPE treatment. The numbers of IL-17A producing cells in the lung were also increased following PPE treatment. Thus, elastase administration leads to increases in both IL-17-producing cells and levels of IL-17A. These results were consistent with the observations showing that IL-17A expressing cells were increased in the airways of COPD patients. For example, numbers of IL-17A+ cells in the bronchial submucosa were increased in COPD compared to smokers without COPD and nonsmoking control subjects [19]. The sputum concentrations of IL-17A in COPD were increased compared to patients with asthma [19]. It has also been reported that cigarette smoke extract had an adjuvant effect on CD4 T cell activation and differentiation to Th17 cells in vitro [38]. These data suggest that smoking induces both IL-17A secretion in the airways and increases differentiation of Th17 cells.

IL-17A has been shown to accumulate [39] and activate neutrophils [40], and enhance epithelial production of IL-8 [41]. It has also been reported that IL-17A acts directly on epithelial cells, airway fibroblasts and smooth muscle cells to induce the secretion of neutrophil-recruiting chemokines [42]. Airway smooth muscle strips from COPD patients expressed IL-17RA and responded to IL-17A by inducing IL-8 production [16]. Rodents lack a direct homologue of IL-8, but the chemokines KC and MIP-2 are regarded as functional homologues and have been found to contribute to the pathology of a number of neutrophil-dependent animal models of disease [43]. In the present study, levels of KC and MIP-2 were increased in WT mice following PPE treatment, and these levels were lower in IL-17A−/− mice. Thus IL-17A may induce neutrophil chemotactic factor production, KC and MIP-2, and contribute to neutrophilic airway inflammation following PPE treatment.

Th17 cells have been shown to produce more inflammatory cytokines, such as IL-1β, compared with regulatory T cells and other T-helper cells [44]. In the present study, the levels of IL-1β in WT mice were increased 4 days after intratracheal PPE instillation and levels were significantly lower in IL-17A−/− mice. Although the link between acute inflammatory changes and the subsequent development of emphysematous changes requires further investigation, it is likely that in addition to KC and MIP-2, increased levels of proinflammatory cytokines such as IL-1β prolong and enhance acute inflammation and contribute to later emphysema. In this way, IL-17A-mediated pathways might play a central role in the pathophysiological events.

Chen et al. recently reported that IL-17RA-deficient mice failed to develop emphysema after 6 months of cigarette smoke exposure in contrast to wild-type mice [38]. In their study, the wild-type mice which received 6 months smoking exposure showed higher levels of IL-17 in BAL fluid compared to sham-exposed mice. IL-17RA−/− mice showed lower levels of monocyte chemotactic protein-1 (MCP-1) in BAL fluid and lower expression of the matrix metalloproteinase (MMP)-9 and MMP-12 in the lung, compared to wild-type mice, suggesting an important role for IL-17RA in macrophage recruitment to the inflamed lung and for development of emphysema. They saw no differences in KC levels in BAL fluid between IL-17RA−/− mice and wild-type mice when emphysema was established, similar to our study. In the present study, however, we have shown that neutrophil-related chemokines such as KC and MIP-2 were increased at a relatively early stage following elastase treatment, which was associated with increased levels of IL-17A, and increased numbers of neutrophils in the airways, suggesting an important role for IL-17A in the recruitment of neutrophils at a relatively early stage. IL-17RA has at least three ligands including IL-17A, IL-17 F, and IL-25. In the present study, we demonstrated the importance of IL-17A for emphysema using IL-17A−/− mice. Shan et al. have also shown that cigarette smoke-induced emphysema is mediated by IL-17A in mice [45]. Regarding other ligands for IL17RA, IL-25 has been reported to be associated with both Th2 and Th17 immune responses [46], however, little is known about the role of this cytokine in emphysema development. Expression of both IL-17A and IL-17 F were increased in the airways of COPD subjects in both inflammatory cells as well as in the airway epithelium [18,19]. In contrast, numbers of IL-17 F+ cells were not increased in COPD compared to control subjects [20].

In the present study, elastase-induced emphysematous changes of the lung in IL-17A−/− mice were reduced, but not completely attenuated compared to WT mice, suggesting that IL-17A may be essential but not sufficient for the full development of elastase-induced emphysema. Previous studies have shown that Th1 cells contribute largely to the inflammatory cascade of events related to the pathogenesis of COPD [34,35]. It has also been reported that Th2 cells may be important in the inflammatory response of the disease [47-49]. Using IL-18 transgenic mice with disruption of the IL-13 gene, Hoshino et al. have shown that IL-18 and IL-13 might have important roles in the pathogenesis of emphysema [50]. Recently, it has also been reported that overexpression of IL-18 induced emphysema via IFN-γ and IL-17A, and induced mucus metaplasia via IL-13 [51]. Chronic cigarette smoke has been shown to induce both Th1 and Th17 cells in mice [52]. Thus, although Th17 cells may play an important role in the pathogenesis of emphysema, their role is not exclusive and Th1 and Th2 cells may also have contributory roles. Further understanding of how these helper T cell subsets interact and orchestrate a pathological response to smoking will be essential if we are to successfully intervene in the development and progression of COPD and emphysema.

## Conclusions

We demonstrated that IL-17A plays an important role to the development of PPE-induced acute phase neutrophilic inflammation and subsequent emphysematous change of the lung, which was associated with increased levels of neutrophil-related chemokines such as KC, MIP-2 and IL-1β. Numbers of Th17 cells as well as IL-17A levels in the lung were increased following treatment with elastase. Our data suggest that targeting IL-17A in emphysema may be a hopeful therapeutic strategy for improving clinical outcomes and for delaying disease progression (Additional file 1).

## Abbreviations

BAL: Bronchoalveolar lavage; Cst: Static compliance; COPD: Chronic obstructive pulmonary disease; KC: Keratinocyte-derived chemokine; Lm: The mean linear intercept; MCP-1: Monocyte chemotactic protein-1; MIP-2: Macrophage inflammatory protein 2; MMP: Matrix metalloproteinase; PPE: Porcine pancreas elastase; Th: T-helper.

## Competing interests

None of the authors have any conflicts of interest, financial or non-financial, to disclose.

## Authors’ contributions

EK, KW, AT, GI, HK, and YI carried out the animal experiments. EK and HN carried out the flow cytometory. EK and AK performed the statistical analysis. NM, AK, HN, YT, MK, YI, EWG, and MT helped to draft the manuscript. NM conceived of the study, and participated in its design and coordination. All authors read and approved the final manuscript.

## Financial support

The project described was supported in part by a grant from the Ministry of Education, Science and Culture of Japan.

## Supplementary Material

Additional file 1 **Figure S1. Cellular composition in BAL fluid on day 2 after intratracheal instillation of PPE or saline.** *p<0.05 compared to WT saline and IL-17A-/- saline. #p<0.05 versus WT PPE. n=8 in each group. Click here for file
