# Supplementary material for: IL-17A is essential to the development of elastase-induced pulmonary inflammation and emphysema in mice
Source: Respir Res. 2013 Jan 20;14(1):5. doi: 10.1186/1465-9921-14-5 (PMC3564829; doi:10.1186/1465-9921-14-5)
Supplement: Additional file 1 — Figure S1. Cellular composition in BAL fluid on day 2 after intratracheal instillation of PPE or saline. *p<0.05 compared to WT saline and IL-17A-/- saline. #p<0.05 versus WT PPE. n=8 in each group. [file 1465-9921-14-5-S1.pdf]

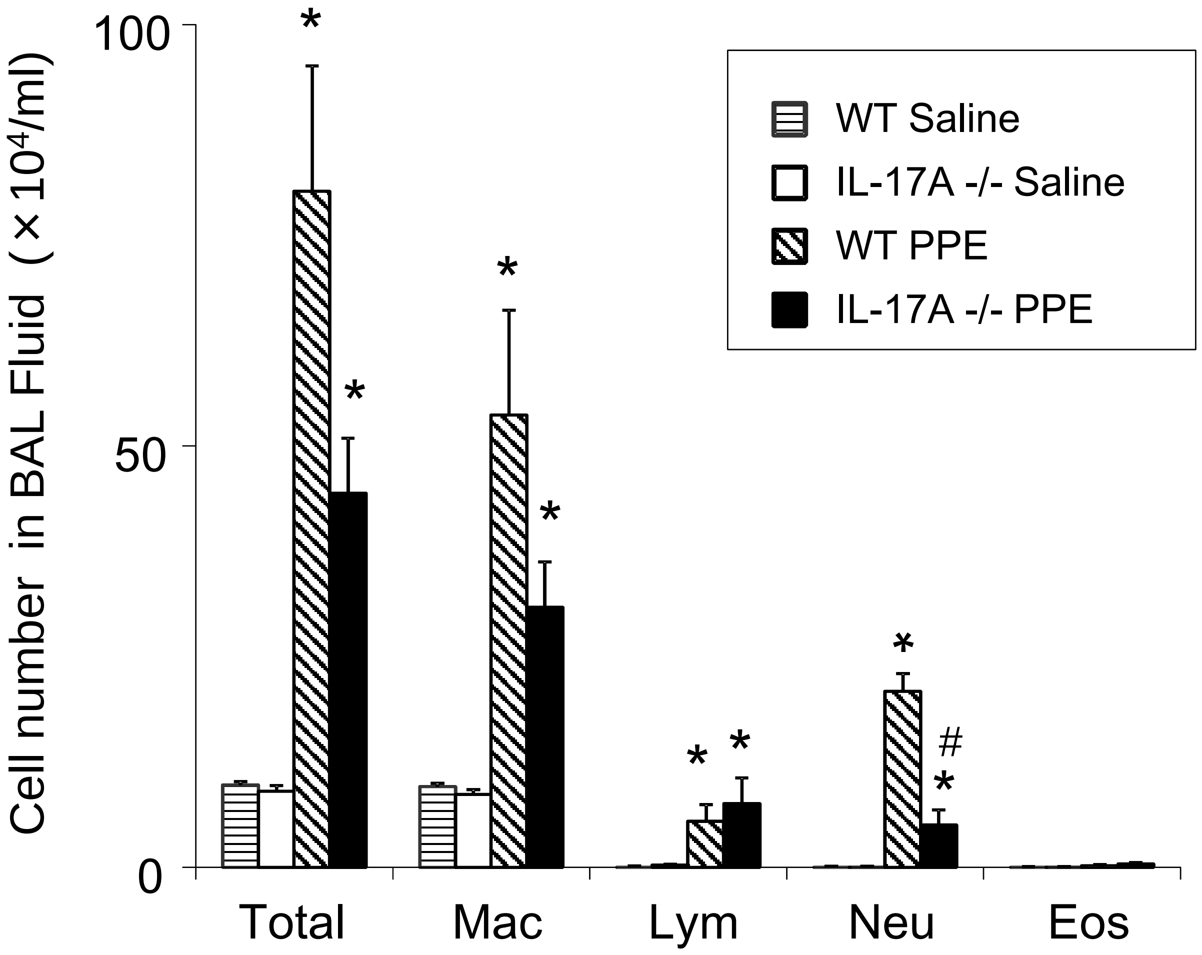

Cellular composition in BAL fluid on day 2 after intratracheal instillation of PPE or saline. *p<0.05 compared to WT saline and IL-17A-/- saline. #p<0.05 versus WT PPE. n=8 in each group.
